# A Warning Instance

**Published:** 1888-03

**Authors:** 


### A WARNING INSTANCE.

If every correspondent of this journal would take particular pains to write all names distinctly it would save some annoying mistakes. It is impossible to determine a proper name from its connection in the sentence, as may be the case with other words. How often have we been led to sympathize with poor, much-abused Belshazzar, of Biblical fame, whose knees smote together when he saw an illegible handwriting which it was essential that he should so construe that it would make sense. We have undergone about the same sensation under similar circumstances, and we were at a disadvantage, too, in having no sharp lawyer at hand who was skilled in interpreting the curious hieroglyphics of some correspondent who appeared to write with his finger rather than with a pen. Will not those who favor the INDEPENDENT PRACTITIONER use a degree of caution that will prevent such addling of editorial brains as may make it necessary to turn us also out to grass ?

